# Histopathological Features of Hepatocellular Carcinoma in Patients with Hepatitis B and D Virus Infection: A Single-Institution Study in Mongolia

**DOI:** 10.3390/cancers17030432

**Published:** 2025-01-27

**Authors:** Orgil Jargalsaikhan, Wenhua Shao, Mayuko Ichimura-Shimizu, Soichiro Ishimaru, Takaaki Koma, Masako Nomaguchi, Hirohisa Ogawa, Shotaro Tachibana, Battogtokh Chimeddorj, Khongorzul Batchuluun, Anujin Tseveenjav, Battur Magvan, Bayarmaa Enkhbat, Sayamaa Lkhagvadorj, Adilsaikhan Mendjargal, Lkhagvadulam Ganbaatar, Minoru Irahara, Masashi Akaike, Damdindorj Boldbaatar, Koichi Tsuneyama

**Affiliations:** 1 Department of Pathology and Laboratory Medicine, Tokushima University Graduate School of Biomedical Sciences, Tokushima 770-8503, Japan; c202156006@tokushima-u.ac.jp (O.J.); c201901052@tokushima-u.ac.jp (S.I.); ogawa.hirohisa@tokushima-u.ac.jp (H.O.); c202356033@tokushima-u.ac.jp (S.T.); tsuneyama.koichi@tokushima-u.ac.jp (K.T.); 2 Department of Molecular Pathology, Tokushima University Graduate School of Biomedical Sciences, Tokushima 770-8503, Japan; shao.wenhua@tokushima-u.ac.jp; 3 Department of Microbiology, Tokushima University Graduate School of Biomedical Sciences, Tokushima 770-8503, Japan; tkoma@tokushima-u.ac.jp (T.K.); nomaguchi@tokushima-u.ac.jp (M.N.); 4 Institute of Biomedical Sciences, Mongolian National University of Medical Sciences, Ulaanbaatar 14210, Mongolia; khongorzul.bat@mnums.edu.mn (K.B.); anujin.ts@mnums.edu.mn (A.T.); 5 Department of Microbiology and Infection Prevention Control, School of Biomedicine, Mongolian National University of Medical Sciences, Ulaanbaatar 14210, Mongolia; battur@mnums.edu.mn; 6 Department of Pathology and Forensic Medicine, School of Biomedicine, Mongolian National University of Medical Sciences, Ulaanbaatar 14210, Mongolia; bayarmaa.e@mnums.edu.mn (B.E.); sayamaa@mnums.edu.mn (S.L.); 7 Surgery Department, Mongolia–Japan Hospital of Mongolian National University of Medical Sciences, Ulaanbaatar 14210, Mongolia; adilsaikhan@mnums.edu.mn (A.M.); lkhagvadulam.g@mnums.edu.mn (L.G.); 8 Department of Obstetrics and Gynecology, Tokushima University Graduate School of Biomedical Sciences, Tokushima 770-8503, Japan; irahara@tokushima-u.ac.jp; 9 Department of Medical Education, Graduate School of Biomedical Sciences, Tokushima University Graduate School of Biomedical Sciences, Tokushima 770-8503, Japan; akaike.masashi@tokushima-u.ac.jp; 10 Department of Physiology, School of Bio-Medicine, Mongolian National University of Medical Sciences, Ulaanbaatar 14210, Mongolia; damdindorj@mnums.edu.mn

**Keywords:** hepatocellular carcinoma, chronic hepatitis, HDV, HBV, CD4, CD8

## Abstract

**Background**: Viral hepatitis, particularly hepatitis B (HBV) and hepatitis C (HCV), is highly prevalent in Mongolia. Moreover, Mongolia has the highest prevalence of hepatitis delta virus (HDV) globally, with over 60% of HBV-infected individuals also co-infected with HDV. Since HBV/HDV infections accelerate liver disease progression more compared to HBV infection alone, urgent national health measures are required. **Method**: This study presents a clinicopathological analysis of 49 hepatocellular carcinoma cases surgically resected at the Mongolia–Japan Hospital of the Mongolian National University of Medical Sciences. **Results**: HBV infection was found in 27 (55.1%) cases of all HCC cases. Immunohistochemical staining of the liver revealed that 14 (28.6%) cases were HDV antigen-positive in the HCC cases. HDV-positive cases exhibited significantly higher inflammatory activity compared to HDV-negative cases, with lymphocytic infiltrates predominantly composed of CD4-positive cells. Furthermore, HDV-positive cells were spatially distinct from HBs antigen-positive cells, suggesting that HDV-infected cells may interfere with HBV replication. No significant differences in fibrosis or in tumor characteristics were observed between the HDV-positive and negative cases. Early diagnosis of HBV/HDV infections is essential for appropriate treatment and to prevent further domestic transmission of the virus. However, routine testing for HDV infection is rarely conducted in Mongolia. Since HDV-positive cells are morphologically indistinguishable from surrounding HDV-negative cells, routine histopathological analysis may not be sufficient enough to detect HDV infection. **Conclusions**: Based on this clinicopathological study, CD4 and CD8 immunostaining can be considered an adjunctive diagnostic tool in cases with significant lymphocytic infiltration and hepatocellular damage. Additionally, HDV screening using blood and tissue samples may be recommended to ensure accurate diagnosis.

## Simple Summary

Mongolia has a high prevalence of viral hepatitis, particularly hepatitis B virus (HBV), with an estimated 60% of HBV-infected individuals co-infected with hepatitis D virus (HDV), which accelerates liver disease progression. However, there is limited data on the contriburion of HDV infection to hepatocellular carcinoma (HCC) in Mongolia. This study clinicopathologically analyzed 49 HCC cases from the Mongolia–Japan Hospital between August 2020 and July 2024. Among these, 55.1% had HBV, and 28.6% were HDV-positive. Including the hepatitis C virus, a total of 75.5% of cases are viral hepatitis-related HCC. HDV-positive HCC cases showed marked inflammation in the non-cancerous liver tissue with infiltration of CD4-positive T-cells, with fewer CD8-positive cells. This study shows the current situation of HDV-related HCC in Mongolia and also suggests the usefulness of using CD4/CD8 immunostaining to determine HDV infection histologically in regions where routine testing for HDV is rare.

## 1. Introduction

In individuals with chronic hepatitis B virus (HBV) infection, superinfection with hepatitis D virus (HDV) is known to result in a higher likelihood of severe disease progression compared to HBV infection alone. HDV possesses a circular, single-stranded RNA genome that replicates using host cellular machinery rather than its own viral polymerase unlike most animal RNA viruses. This replication process, coupled with its requirement for the hepatitis B surface antigen (HBsAg) for infectivity, underscores the complex interplay between HDV and its host [1]. Although the primary oncogenic mechanisms of HBV are known to involve frequent integration of the viral genome and TP53 mutations, HDV superinfection suppresses HBV replication [2]. Despite this suppression, liver pathology becomes more severe compared to HBV infection alone. In 2018, Diaz et al. analyzed gene expression profiles in hepatitis virus-associated HCC, including cases with HDV. Their findings revealed that tumor tissues from HDV-superinfected individuals exhibit a unique molecular profile, distinct from HBV-only cases, characterized by upregulation of genes involved in the cell cycle, DNA replication, DNA damage, and DNA repair [3]. This suggests that the oncogenic mechanisms differ between HDV-superinfected and HBV-only infections. HBV primarily contributes to hepatocellular carcinoma (HCC) initiation through the integration of its DNA into the host genome, disrupting, genetic stability and activating oncogenes. Chronic HBV infection exacerbates hepatocyte turnover and inflammation, creating a pro-oncogenic environment. HDV intensifies these effects by driving more severe liver inflammation and fibrosis, accelerating disease progression [4,5].

Liver disease in Mongolia presents a significant public health challenge, with incidence rates of liver cancer and cirrhosis among the highest globally. A key factor behind this is the high prevalence of HBV and HDV co-infection among HBV carriers, which accelerates the progression of liver disease and increases the risk of hepatocellular carcinoma (HCC) [6,7,8,9]. In Mongolia, more than 60% of HBV patients are also infected with HDV, being markedly higher than the global HBV/HDV infection rate of approximately 13% [10,11,12].

The HBV vaccine, introduced in the 1980s, remains the most effective preventive measure against HBV. The World Health Organization (WHO) recommends administering the first dose within 24 h of birth, followed by subsequent doses according to national immunization schedules [13]. This approach has significantly lowered HBV infection rates worldwide, particularly in high-risk regions such as Asia and Africa. For example, national vaccination campaigns in China and Taiwan have markedly reduced HBV transmission among younger populations [14,15]. Similarly, Mongolia has adopted HBV vaccination programs, leading to a decrease in infection rates among younger demographics. However, challenges persist, especially in rural areas where knowledge about hepatitis is limited, leading to continued intra-family transmission from untreated carriers [16,17]. In contrast to HBV, no vaccine is currently available for the hepatitis C virus (HCV). As HCV is primarily transmitted via blood, stringent infection control measures are crucial, particularly in medical settings. These include the use of disposable syringes, appropriate sterilization protocols, and rigorous testing of blood products. In many countries, harm-reduction initiatives, such as providing clean needles to intravenous drug users, have proven effective in reducing HCV transmission [18]. Furthermore, regular screening, particularly in high-risk groups, such as injection-drug users and those who have received blood transfusions, is critical for early detection and intervention.

To mitigate the impact of liver disease, Mongolian government initiatives such as the “Healthy Liver” campaign aim to raise public awareness and improve access to antiviral treatments [19]. To assess the impact of these efforts and better understand the epidemiological and clinical landscape of liver disease in Mongolia, further investigation is needed.

This study examines 49 recently resected HCC patients from the Mongolia–Japan Hospital of the Mongolian National University of Medical Sciences (MNUMS) in Ulaanbaatar. Through clinical-pathological assessment and immunohistochemical analysis for HDV antigens, we aimed to elucidate the morphological features of HBV/HDV infections and provide insights into the current state of liver cancer in Mongolia.

## 2. Materials and Methods

### 2.1. Selection of Cases

Seventy-two liver cancer patients who underwent surgical resection were extracted from medical records of the Mongolia–Japan Hospital of MNUMS in Ulaanbaatar between August 2020 and July 2024. All patients were initially assessed by a Mongolian pathologist (S.L.), and 22, including those with cholangiocarcinoma and hemangioma, were excluded. One patient with squamous cell carcinoma was excluded after re-evaluation by a Japanese liver pathologist (K.T.). Thus, a total of 49 HCC patients were analyzed (age: 65 [IQR: 55, 69]; sex: male 27, female 22). For each patient, clinical data, including sex, age, liver function, blood biochemistry, and viral infection status, were collected. The virus status was determined by commercially available ELISA. The data of treatment status and viral loads in blood could not be obtained in the present study.

### 2.2. Histological Analysis

Histopathological evaluation of hematoxylin and eosin (HE)-stained slides was performed, with slides selected from both cancerous and non-cancerous liver tissue (background liver). Consensus evaluation of liver pathology was performed by three expert liver pathologists (J.O., S.W., and K.T.). To assess tumor histology, inflammatory activity was graded on a 4-point scale (A0: no activity, A1: mild activity, A2: moderate activity, and A3: severe activity), and the fibrosis stage was assessed on a 5-point scale (F0: no fibrosis, F1: portal fibrosis without septa, F2: portal fibrosis with few septa, F3: numerous septa without cirrhosis, and F4: cirrhosis) based on the METAVIR score [20]. If there was notable variability in activity or fibrosis between different regions of the liver, a mixed score (e.g., A1–A2) was assigned, and the median value was used for statistical purposes.

HCC characteristics were also evaluated, focusing on the presence of clear cells, fat deposition, significant fibrosis, and vascular invasion. Each of these features was scored on a binary scale (presence or absence). The degree of lymphocytic infiltration within and around the tumor (intra- and peritumoral) was classified as either prominent or not prominent.

### 2.3. Immunohistochemistry

Immunohistochemical staining was performed in all patients from the Mongolia–Japan Hospital of MNUMS. Antibodies targeting hepatitis B surface antigen (HBsAg) and HDV antigens (both large and small delta antigens) were used. After deparaffinization of the specimens, antigen retrieval was performed with microwave heating. Following the application of primary antibodies (mouse monoclonal anti-HBs) Ab. [5C3] (GeneTex, Irvine, CA, USA) and rabbit monoclonal-anti HDV (large and small delta antigens) Ab. (Abcam, Cambridge, UK), secondary antibodies against mouse/rabbit IgG with peroxidase (Envision-PO for mouse or rabbit, Agilent, Santa Clara, CA, USA) were applied. The diaminobenzizine (DAB) reaction (Abcam) was conducted for the color of peroxidase. Counterstaining by hematoxylin was performed. Five patients who tested positive for the HDV antigen and who showed prominent lymphocytic infiltration were selected for double-immunostaining using mouse monoclonal anti-CD4 Ab. (NCL-CD4-1F6) (Leica Biosystems, Milton Keynes, UK) and mouse monoclonal anti-CD8 Ab. (Leica Biosystems) to assess the immune response within liver tissue. Primary (CD4) and secondary (Envision-PO) antibodies were applied using the standard enzyme-antibody method, with CD4-positive cells labeled in brown using DAB as the substrate. The specimen was then immersed in PBS at a temperature of 95 °C or higher for 15 min to inactivate the initially applied primary and secondary antibodies. CD8 antibodies and Envision-PO were subsequently applied, and CD8-positive cells were labeled in blue using the chromogenic substrate PermaBlue/HRP (Diagnostic Biosystems, Pleasanton, CA, USA).

### 2.4. Statistical Analysis

All data are expressed as median (interquartile range), or frequency (percentages) as applicable. Liver histopathological features with HDV infection were compared between the two patient groups who were positive or negative for HDV antigens. The HCV co-infection patients included one patient each in the HDV-positive and negative groups. The Mann–Whitney U test was applied to compare continuous variables between the two groups. The chi-square test and Fisher’s exact test were used to evaluate any associations between categorical variables. A *p*-value of less than 0.05 was considered significant. All tests were conducted using IBM SPSS 29.0 (IBM, Armonk, NY, USA).

## 3. Results

### 3.1. Clinical and Pathological Characteristics of HCC Patients

**Figure 1:**
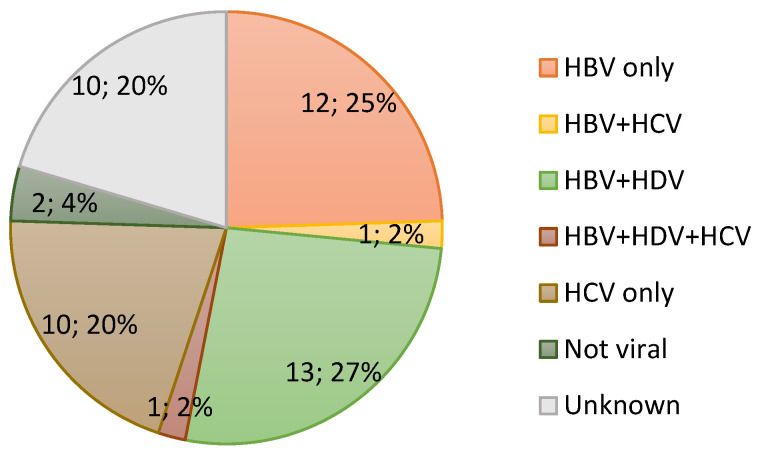
The rates of viral hepatitis in HCC patients.

Comprehensive clinical data were available for most patients from the Mongolia–Japan Hospital of MNUMS, although serological data, particularly regarding the HDV status, were confirmed for only a few (Supplemental Table S1). Therefore, HDV infection was defined as being positive for the HDV antigen by immunohistochemical assessment instead of by serological diagnosis in the present study. HBV infection was judged based on either serological diagnosis or immunohistochemical evaluation. HBV infection was identified in 27 (55.1%) among all HCC patients (Figure 1). Twelve and fifteen cases were diagnosed as HBV-positive by serological and immunohistochemical methods, respectively (Supplemental Table S1). HDV Ag-positivity was noted in the liver of 14 (28.6%) patients, with one of these patients co-infected with HCV. Thus, HDV was present in 51% of patients with HBV-related HCC. The etiology of 12 patients was unknown, as the viral status could not be evaluated due to lack of formalin-fixed paraffin-embedded blocks. None of the patients exhibited signs of autoimmune hepatitis or primary biliary cholangitis. Although mild-moderate steatosis was observed in nine patients, there was no histological evidence of steatohepatitis, such as ballooning or perivenular-pericellular fibrosis. Therefore, it was considered that most cases of HCC were mainly related to viral hepatitis.

The rates of viral hepatitis based on liver histology or serology among patients who had surgery for HCC. The numbers in the pie chart indicate the number of cases.

### 3.2. Pathological Features of Background Liver Tissue in HDV-Positive Patients

**Figure 2:**
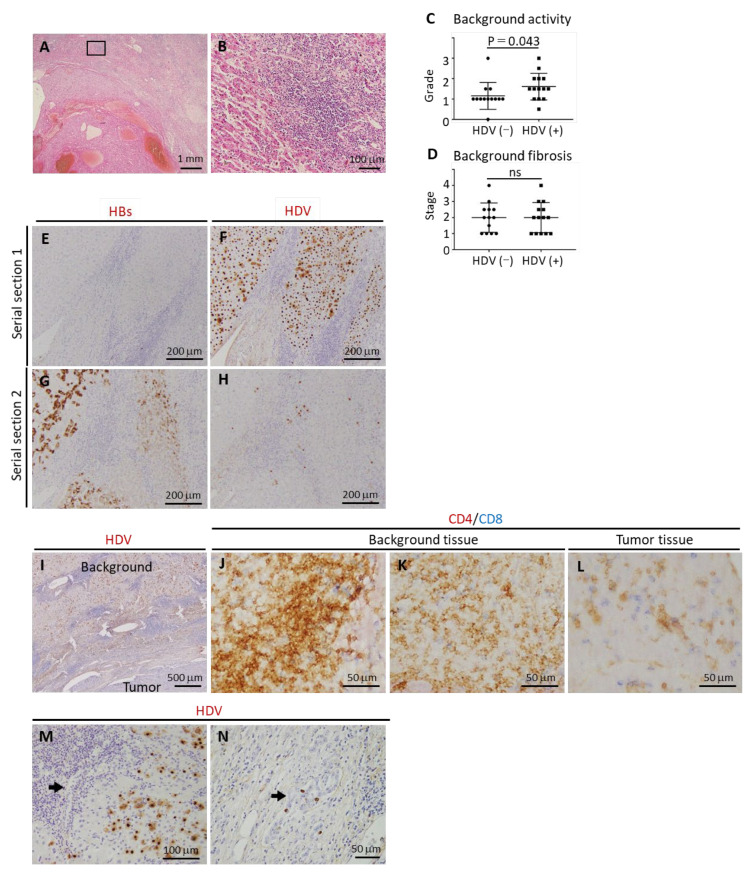
Pathological features of background liver tissue in HDV-positive HCC patients. (**A**,**B**): Representative images of HE staining in HDV-positive patients. (**A**): Low-power view showing both the tumor and surrounding (background) liver tissue. Small regenerative nodules are visible around the primary encapsulated nodule. The surrounding liver tissue shows significant inflammatory cell infiltration, accompanied by advanced fibrosis. The region enclosed by the rectangle is shown at higher magnification in panel (**B**). (**B**): Higher magnification of the background liver tissue. Marked infiltration of lymphocytes is present, particularly at the interface, with clear evidence of hepatocyte damage due to the immune response. (**C**,**D**): Comparative analysis of inflammation and fibrosis scores in the background liver of HDV-positive and -negative HBV-infected HCC patients. Inflammatory activity was significantly elevated in HDV-positive patients (*p* = 0.043). (**E**,**G**): HBsAg immunostaining. (**F**,**H**): HDV immunostaining. (**E**,**F**) represent serial sections of the same case, as do (**G**,**H**). In (**E**,**F**), only a few HBsAg-positive cells are present, while a large number of HDV-positive cells can be noted. In contrast, (**G**,**H**) show clusters of HBsAg-positive cells, but only a few HDV-positive cells. Notably, there is minimal overlap between HBsAg-positive and HDV-positive cells. (**I**–**L**): Lymphocytic profile surrounding HDV-positive hepatocytes. (**I**): HDV immunostaining in the background liver shows widespread HDV-positive hepatocytes. Significant lymphocytic infiltration is observed, particularly at the interface hepatitis regions in portal areas and within fibrous septa. In contrast, HDV-positive cells are nearly absent within the tumor. (**J**–**L**): Double immunostaining of lymphocytes (CD4 in brown, CD8 in blue) infiltrating the liver. (**J**,**K**): In the background liver, the majority of infiltrating lymphocytes are CD4-positive, with only a few CD8-positive cells. (**L**): In the tumor, lymphocytic infiltration shows a more balanced ratio of CD4-positive and CD8-positive cells. (**M**,**N**); Immunohistochemical detection of HDV antigen in the biliary system. (**M**): HDV-positive cells were confined to hepatocytes, with no HDV antigen expression observed in the interlobular bile ducts (arrow). (**N**): In one patient, an HDV antigen was detected in the nuclei of proliferating bile ductules (arrow) located in the interface region with marked inflammation.

The histopathological characteristics of HCC patients are summarized in Supplemental Table S2. Figure 2A shows typical histological images of the tumor and background liver in HDV-positive patients. In the background liver, marked lymphocytic infiltration was observed in fibrous septa showing severe interface hepatitis (Figure 2B). Hepatocytes were damaged by infiltrating lymphocytes. Fibrous extension occurred from the portal area to varying extents. In HBV-related HCC, inflammatory activity in the background liver was notably elevated in HDV-positive patients as compared with HDV-negative patients (Figure 2C, *p* = 0.043), although no significant difference in fibrosis severity was identified between the groups (Figure 2D).

Immunohistochemical analysis demonstrated distinct segregation between HBsAg-positive and HDV antigen-positive regions. HBsAg-positive cells were sparse in areas with diffuse HDV positivity; conversely, HDV-positive cells were rarely present in regions with clustered HBsAg-positive cells (Figure 2E–H). Morphologically, no cytoplasmic inclusions or other distinguishing features were observed by HE staining in areas containing HDV-positive cells. Notably, there was significant lymphocytic infiltration, particularly with marked interface hepatitis surrounding HDV-positive hepatocytes in the background liver (Figure 2I). These infiltrating lymphocytes were predominantly CD4-positive T cells, with fewer CD8-positive cells (Figure 2J,K). In contrast, an equal distribution of CD4-positive and CD8-positive cells was observed within tumors that lacked HDV-positive cells (Figure 2L). HDV antigen expression was confined to hepatocytes and absent from interlobular bile ducts, except in one patient in whom HDV antigen was detected in the nuclei of proliferating bile ductules (Figure 2M,N).

### 3.3. Pathological Features of Hepatocellular Carcinoma in HDV-Positive Patients

**Figure 3:**
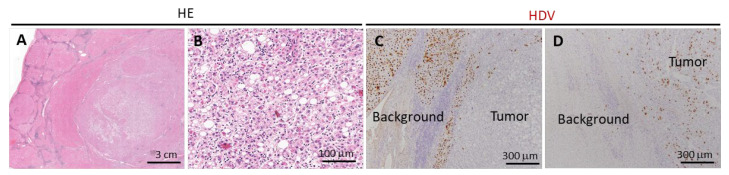
Characteristics of liver tumors in HDV-positive patients. (**A**,**B**): Representative images of liver tumors (**A**): low magnification, (**B**): high magnification of the tumor center). (**C**,**D**): HDV immunohistochemistry. Most hepatocellular carcinomas (HCCs) showed only a small number of HDV-positive cells (**C**). However, in two patients, the number of HDV-positive cells within tumor tissue exceeded that in surrounding liver tissue (**D**).

HCCs in this study predominantly presented as simple nodular types, with a frequent nodule-in-nodule growth pattern (Figure 3A,B). Tumors typically contained a mix of well and moderately differentiated components, with poorly differentiated or undifferentiated elements being rarely observed (Supplemental Table S2). Tumor characteristics, such as clear cell type, fat deposition, intratumoral fibrosis, lymphocytic infiltration (both intra- and peritumoral cuffing), and vascular invasion, did not show significant differences between HDV-positive and HDV-negative patients. However, there was a trend towards increased fat deposition within the tumor tissue in HDV-positive patients, although this difference did not reach significance (*p* = 0.066, Table 1). While most HCCs contained only a few HDV antigen-positive cells (Figure 3C), in two patients, the number of HDV-positive cells was higher within the tumor tissue compared with the surrounding liver tissue (Figure 3D).

## 4. Discussion

HDV, a satellite virus reliant on HBV, causes the most severe form of chronic hepatitis and is correlated with significant morbidity and mortality [21]. The clinical spectrum of HDV infection ranges from asymptomatic to acute liver failure and advances to chronic hepatitis, with rapid progression to cirrhosis, liver failure, or HCC, exceeding the severity typically observed with HBV infection alone. Although HBV/HDV infections can sometimes resolve spontaneously, superinfection of chronic HBV carriers with HDV almost invariably leads to chronic HDV infection [4,22].

The assembly and release of virus particles, as well as HDV entry into hepatocytes, depend on the presence of HBV S protein. However, HBV is not required for HDV replication, as HDV can replicate in the nucleus by interacting with host proteins [21]. Chronic hepatitis D is characterized by a severe and progressive disease course, with cirrhosis developing in up to 80% of cases, often culminating in liver failure or HCC [23,24]. HDV exacerbates HBV-associated liver pathology, accelerating processes such as inflammation, fibrosis, and cirrhosis. Recent research indicated that HDV enhances oxidative stress and endoplasmic reticulum (ER) stress caused by HBV [25].

In the present study, viral hepatitis accounted for most HCC patients in Mongolia, with HDV considered to be involved in approximately one-third of cases. Histological analysis revealed moderate to severe lymphocytic infiltration around destroyed HDV-positive hepatocytes, suggesting that immune responses to HDV antigens may contribute significantly to the severity of HDV-associated liver disease [26]. Notably, HDV-positive patients exhibited increased lymphocytic infiltration, particularly involving CD4+ rather than CD8+ T cells. This finding is consistent with previous reports describing predominant infiltration of cytotoxic CD4+ T cells expressing perforin around HDV-infected cells [25]. The presence of active inflammation and fibrosis, particularly interface hepatitis dominated by CD4+ T cells, may indicate HBV/HDV infections. The complex interplay between the immune response and viral evolution in chronic HDV infection is notable. While the immune response to HDV is heavily mediated by CD8+ T cells, the data also suggest a significant role for CD4+ T cells in the presence of persistent infection. This impaired response may be a consequence of viral mutations that allow HDV to escape CD8+ T-cell recognition, as discussed in studies where HDV variants evolve to avoid detection by common HLA class I molecules. These immune escape mechanisms reduce the effectiveness of CD8+ T-cell responses, potentially leading to an increased involvement of CD4+ T cells in the immune response. Predominant infiltration of cytotoxic CD4+ T cells expressing perforin in the liver could compensate for the diminished CD8+ T-cell function and target HDV-infected cells. The shift toward CD4+ T-cell involvement may reflect an adaptive immune strategy as the virus evades typical CD8+ T-cell-mediated clearance. Additionally, chronic viral infections induce exhaustion in virus specific CD8+ T cells, with recovery from this exhaustion a key therapeutic goal. The functional support provided by CD4+ T cells is believed to compensate for this exhaustion, highlighting their critical role in disease outcomes [27]. Understanding these immune dynamics is crucial for developing new therapeutic approaches that aim to restore effective CD8+ T-cell responses and harness the role of CD4+ T cells in controlling chronic HDV infection [28,29].

Interestingly, in HDV-positive cases, HBsAg-positive cells and HDV antigen-positive cells were spatially distinct, suggesting that HDV may interfere with HBV replication. Previous research demonstrated that hepatitis delta antigen (HDAg) directly repressed HBV enhancer activity [30]. The small form HDAg, one of the HDAg encoded by HDV, inhibits the binding transcription factors signal transducer and activator of transcription 3 and hepatocyte nuclear factor to the HBV enhancers, while the large form HDAg modulates HBsAg synthesis for HDV assembly. HDV also competes with HBV for HBsAg, which is essential for HDV particle assembly, limiting the ability of HBV to form new virions. Additionally, HDV infection triggers strong type I interferon responses, leading to the activation of interferon-stimulated genes that suppress viral replication [31]. Our observation has important implications regarding therapeutic strategies targeting both HBV and HDV infections.

Concerning hepatocarcinogenesis, it has been proposed that the mechanisms by which HBV and HDV contribute to tumor development differ [22]. In the majority of cases, the numbers of HBs-positive and HDV-positive cancer cells were reported to be markedly lower than in the surrounding liver tissue, suggesting that both viruses may promote tumor initiation rather than progression. However, in two HDV-positive patients, the number of HDV-positive cancer cells was significantly higher than in the surrounding background hepatocytes, indicating the possibility of HDV replication within tumor cells. Future studies involving genetic analyses using microdissection systems are warranted to further elucidate the virological status in cancer cells in these cases.

The limitation of the present study is the insufficient data on treatment history and viral load. The differences in serum viral load and treatment methods between HBV-alone and HBV/HDV co-infected cases are unclear, which implies that our results represent a comprehensive histopathology of HDV-related HCC. The lack of an observed association between HDV infection and severity of liver fibrosis in the present study, which contrasts with the previous research findings [16,17], may be attributable to the influence of therapeutic interventions. However, this study is an exploratory investigation into the reality of HDV-related HCC in Mongolia. Future prospective studies will include detailed clinical data, aiming to clarify the direct involvement of HDV. Another unresolved issue is the nuclear expression of HDV antigen in proliferating bile ductules. In this study, no other HDV expression was observed in the biliary system, and no cases including differentiation of cholangiocarcinoma were identified. Proliferating bile ductules are considered to originate either from precursor/stem cells or metaplasia of mature hepatocytes during periods of active interface hepatitis. It is plausible that HDV-positive proliferating bile ductules represent metaplasia from HDV-infected hepatocytes.

The reason for the markedly high prevalence of HBV/HDV infections in the Mongolian population remains unclear. Therefore, identifying factors contributing to this phenomenon is a critical priority for controlling HDV in Mongolia. There is a large difference between the medical environment of rural and urban areas in Mongolia, and there are still rural areas providing insufficient education regarding infectious diseases. Access to treatment for hepatitis in Mongolia remains limited due to a lack of screening, clinical services, and the high cost of antiviral drugs. While the price of medications for hepatitis B and C has dropped by nearly 90%, they still remain too expensive for the majority of Mongolians [32,33]. In addition, the recent discovery of HDV-like viruses in various animal species, including rodents and snakes, challenges the previous understanding of HBV as the sole helper virus for HDV replication. Given the common consumption of wildlife, such as marmots, in Mongolia, animal vectors may play a role in HDV transmission [34]. Comprehensive epidemiological studies are needed to explore the potential involvement of animal reservoirs in HDV transmission.

## 5. Conclusions

This study underscores the critical role of HBV/HDV co-infection in HCC in Mongolia. HDV-positive cases showed heightened inflammatory activity and CD4+ lymphocytic infiltration, emphasizing the immune response’s contribution to liver damage. The spatial segregation of HDV and HBsAg suggests potential interference of HDV with HBV replication.

Routine HDV screening and immunostaining are recommended for accurate diagnosis, alongside improved access to antiviral treatments. These findings highlight the urgent need for targeted public health strategies to mitigate the burden of HBV/HDV-related HCC in Mongolia.

## Figures and Tables

**Table 1:** Observed number of cases with lipid droplet in the liver tumor.

	Lipid Droplet in the Tumor		Total
	Absent	Present	
HDV-positive	11	2	13
HDV-negative	7	7	14
Total	18	9	27

*p* = 0.066 by Fisher’s exact test.

## Data Availability

The data presented in this study are available upon reasonable request to the corresponding author.

## Supplementary Materials

The following supporting information can be downloaded at: https://www.mdpi.com/article/10.3390/cancers17030432/s1, Supplemental Table S1: Individual clinical data on HCC patients, Supplemental Table S2: Individual histopathological features of HCC patients.
