# Supplementary material for: Histopathological Features of Hepatocellular Carcinoma in Patients with Hepatitis B and D Virus Infection: A Single-Institution Study in Mongolia
Source: Cancers (Basel). 2025 Jan 27;17(3):432. doi: 10.3390/cancers17030432 (PMC11815750; doi:10.3390/cancers17030432)
Supplement: Supplementary file 1 [file cancers-17-00432-s001.zip › cancers-3420226-supplementary.pdf]

Supplemental Table S1. Individual clinical data on HCC patients.

No.	Age	Sex	Blood biochemistry				Viral infection			Immunohistochemistry	
			WBC (10^9/L)	ALT (U/L)	AST (U/L)	Glucose (mmol/L)	HBV	HDV	HCV	HBs	HDV
1	66	F	7.12	44.37	42.88	4.88	+	ND	+	ND	ND
2	52	F	16.32	128.9	308	ND	ND	ND	ND	-	-
3	53	M	ND	ND	ND	ND	ND	ND	ND	+	-
4	63	M	12.16	128.69	120.92	ND	ND	ND	ND	+	+
5	65	F	6.96	71.1	41.73	5.19	ND	ND	ND	-	-
6	58	M	ND	ND	ND	ND	ND	ND	ND	+	-
7	69	M	ND	ND	ND	ND	ND	ND	ND	+	+
8	74	F	5.67	12.28	18.44	5.22	ND	ND	+	-	-
9	78	F	ND	ND	ND	ND	ND	ND	ND	+	-
10	60	M	ND	ND	ND	ND	ND	ND	ND	+	-
11	65	M	14.44	87.79	165	ND	ND	ND	ND	+	+
12	67	M	23.88	64.47	330.37	ND	+	ND	-	+	+
13	69	F	7.37	7.8	25	ND	ND	ND	ND	-	-
14	57	F	4.49	1.56	28.4	6.25	ND	ND	ND	ND	ND
15	72	F	3.72	40	19.89	ND	ND	ND	ND	+	-
16	54	M	10.09	648	743	ND	ND	ND	ND	ND	ND
17	59	F	3.8	18.62	15.95	5.57	ND	ND	ND	+	-
18	74	M	23.3	48.16	98.79	ND	-	ND	-	-	-
19	55	F	ND	ND	ND	ND	ND	ND	ND	ND	ND
20	64	F	5.6	32.32	22.57	4.42	ND	ND	ND	+	-
21	85	M	9.67	37.42	37.3	ND	-	ND	+	ND	-
22	72	M	5.74	144.3	65.2	ND	-	ND	+	-	-
23	56	M	4.89	507	231	ND	+	ND	-	+	+
24	72	F	6.33	11.42	14.38	5.36	ND	ND	ND	-	-

25	69	M	15.88	21	130	ND	ND	ND	ND	-	-
26	66	M	11.62	173.71	217.42	ND	-	ND	+	ND	ND
27	81	F	4.72	35.8	36.94	ND	-	ND	+	-	-
28	67	M	10.64	21	11.16	ND	-	ND	ND	-	-
29	49	M	5.44	ND	ND	ND	+	ND	ND	+	+
30	54	M	10.09	648	743	ND	ND	ND	ND	+	-
31	53	M	4.79	374	192	4.79	+	ND	-	ND	-
32	65	M	10.68	52	61	5.67	-	ND	+	-	-
33	55	F	ND	ND	ND	ND	+	ND	-	-	-
34	72	F	3.97	74.91	64	4.98	+	+	-	+	+
35	65	M	10.82	12.59	73.16	-	-	ND	+	-	-
36	62	M	4.41	134	107.29	5.44	+	ND	-	+	+
37	68	M	12.16	150	170	5.45	-	ND	+	-	-
38	47	F	9.11	37.91	24.05	4.6	-	ND	+	-	-
39	67	F	8.18	315	168.83	ND	+	ND	-	+	+
40	49	M	6.64	186.47	172.28	ND	+	ND	+	+	+
41	64	M	8.29	19.4	21	ND	+	ND	-	-	-
42	47	M	9.02	46.34	46.24	ND	ND	ND	ND	+	+
43	60	F	4.17	117.32	92.26	4.79	+	ND	-	+	+
44	51	M	4.5	37.38	51.49	ND	ND	ND	ND	+	+
45	65	F	ND	ND	ND	ND	ND	ND	ND	+	+
46	66	F	9.07	33.94	32.33	ND	ND	ND	ND	ND	ND
47	37	F	7.84	11.24	13.36	ND	-	ND	-	ND	ND
48	76	M	6.39	139	210	7.4	-	ND	+	-	-
49	51	F	ND	ND	ND	ND	ND	ND	ND	+	-

ALT, alanine aminotransferase; AST, aspartate aminotransferase; HBs, hepatitis B surface antigen; HBV, hepatitis B virus; HCV, hepatitis C; HDV, hepatitis D virus; ND: not done; WBC, white blood cell

Supplemental Table S2. Individual histopathological features of HCC patients.

Viral infection	No.	Background liver tissue			Tumor tissue							
		Inflammatory activity	Fibrosis	Remarks	Differentiation grade	Tumor clear cells	Tumor fat	Intratumor fibrosis	Vascular invasion	Intratumor lymphocytes	Peritumor-cuffing lymphocytes	Remarks
HBV	3	1	2		mod	-	-	-	-	-	-	
	6	1	2.5		well-mod	+	-	-	-	+	-	
	9	1	2.5		well	-	+	-	-	-	-	
	10	1	1	mild steatosis	well	+	+	-	-	-	-	
	15	1	1		mod	+	-	-	+	+	-	
	17	1	2		well-mod	-	-	-	-	-	+	
	20	0	1.5	steatosis	mod	-	-	-	-	-	-	
	30	1.5	1		mod	-	-	-	+	-	+	
	31	3	2.5		mod	-	-	-	+	+	-	
	33	1	3		mod	-	-	-	-	-	-	
	41	1	1	mild steatosis	mod	-	-	-	-	-	+	
HCV	8	1	1		mod	-	-	+	+	-	+	
	21	1	0		mod	+	-	-	-	+	-	
	22	1	1.5		mod	+	-	-	+	-	-	
	26	2	1.5		mod-poor	-	-	-	+	+	-	
	27	1	1		mod	+	-	-	-	+	-	
	32	2.5	2.5		mod	-	+	+	+	+	-	
	35	1	2.5	steatosis	mod	-	-	+	+	-	-	
	37	1.5	4	mild steatosis	mod	+	+	-	+	-	-	
	38	0.5	0.5	NRH	well-mod	-	-	-	-	+	-	
	48	1	1		mod	-	-	-	-	-	-	

HBV+HCV	1	1	2		well-mod	-	-	-	+	+	-	
HBV+HDV	4	1.5	2.5		well	-	+	-	-	+	-	
	7	1	1		mod	-	+	-	-	-	-	
	11	3	2		mod	+	+	-	+	-	-	
	12	1.5	2		well-mod	-	+	-	-	-	-	Necrosis
	23	0.5	1		mod	-	-	-	-	-	-	
	29	1.5	1	mild steatosis	mod	-	+	-	-	-	+	
	34	2	2.5		well-mod	-	+	-	-	+	-	
	36	2.5	4	mild steatosis	mod	+	-	-	-	+	-	
	39	1	1		mod	+	-	-	+	-	-	
	42	1.5	1		mod	+	-	-	+	-	-	
	43	1	2	mild steatosis	well	-	+	-	-	+	-	
	44	1.5	3		mod	+	-	-	-	-	-	
	45	2	2		well-mod	+	-	-	+	-	-	
	49	1.5	4		mod	+	-	-	-	-	-	
HBV+HCV+HDV	40	2	3		mod	+	-	-	+	-	-	
No virus	18	1	0		mod-poor	-	-	-	+	-	-	
	47	1	1		mod	-	-	-	+	-	-	
Unknown	2	2.5	4		mod	+	-	+	+	+	-	
	5	1	1		mod	+	-	+	+	-	-	
	13	1	3.5		well-mod	+	-	-	-	+	-	
	14	1.5	3	mild steatosis	well-mod	+	+	-	-	+	-	
	16	1	0.5		mod	+	+	-	+	+	-	
	19	1.5	1		mod	-	-	-	+	-	+	
	24	1	3.5		mod	-	-	-	-	-	-	
	25	ND	ND	no background	mod	+	+	+	+	-	+	
	28	2	2.5	steatosis	well-mod	-	-	-	-	+	-	
	46	1	1		mod	-	-	-	-	+	-	

The differentiation of liver tumors was classified into 3 grades: well-differentiated (well), moderately differentiated (mod), and poorly differentiated (poor). When two different grades of differentiation were observed within a tumor, the two grades were listed side by side.

HBV, hepatitis B virus; HCV, hepatitis C; HDV, hepatitis D virus; NRH, nodular regenerative hyperplasia
